# Classification of Chemical Compounds to Support Complex Queries in a Pathway Database

**DOI:** 10.1002/cfg.387

**Published:** 2004-03

**Authors:** Ulrike Wittig, Andreas Weidemann, Renate Kania, Christian Peiss, Isabel Rojas

**Affiliations:** EML Research GmbH Schloss-Wolfsbrunnenweg 33 Heidelberg 69118 Germany

## Abstract

Data quality in biological databases has become a topic of great discussion. To provide high quality data and to deal with the vast amount of biochemical data, annotators and curators need to be supported by software that carries out part of their work in an (semi-) automatic manner. The detection of errors and inconsistencies is a part that requires the knowledge of domain experts, thus in most cases it is done manually, making it very expensive and time-consuming. This paper presents two tools to partially support the curation of data on biochemical pathways. The tool enables the automatic classification of chemical compounds based on their respective SMILES strings. Such classification allows the querying and visualization of biochemical reactions at different levels of abstraction, according to the level of detail at which the reaction participants are described. Chemical compounds can be classified in a flexible manner based on different criteria. The support of the process of data curation is provided by facilitating the detection of compounds that are identified as different but that are actually the same. This is also used to identify similar reactions and, in turn, pathways.


**Comparative and Functional Genomics**
*Comp Funct Genom* 2004; **5**: 156–162.
Published online in Wiley InterScience (www.interscience.wiley.com). **DOI:** 10.1002/cfg.387

**Conference Paper**

### Classification of chemical compounds to support complex queries in a pathway database

Ulrike Wittig*, Andreas Weidemann, Renate Kania, Christian Peiss and Isabel Rojas
*EML Research GmbH, Schloss-Wolfsbrunnenweg 33, 69118 Heidelberg, Germany*

*Correspondence to:
Ulrike Wittig, EML Research GmbH,
Schloss-Wolfsbrunnenweg 33, 69118 Heidelberg, Germany.
E-mail:
Ulrike.Wittig@eml-r.villa-bosch.de*

### Abstract

**Data quality in biological databases has become a topic of great discussion. To provide high quality data and to deal with the vast amount of biochemical data, annotators and curators need to be supported by software that carries out part of their work in an (semi-) automatic manner. The detection of errors and inconsistencies is a part that requires the knowledge of domain experts, thus in most cases it is done manually, making it very expensive and time-consuming. This paper presents two tools to partially support the curation of data on biochemical pathways. The tool enables the automatic classification of chemical compounds based on their respective SMILES strings. Such classification allows the querying and visualization of biochemical reactions at different levels of abstraction, according to the level of detail at which the reaction participants are described. Chemical compounds can be classified in a flexible manner based on different criteria. The support of the process of data curation is provided by facilitating the detection of compounds that are identified as different but that are actually the same. This is also used to identify similar reactions and, in turn, pathways. Copyright © 2004 John Wiley & Sons, Ltd.**

Received: 7 November 2003
Revised: 11 December 2003
Accepted: 23 December 2003

**Keywords:** **pathway databases; data curation; classification; chemical compound**

### Introduction

Much of the current knowledge on biochemical pathways is stored in so-called 'metabolic pathway databases'. The main representatives of these databases are Kyoto Encyclopedia of Genes and Genomes (KEGG: **http://www.genome.ad.jp/kegg/**), EcoCyc (**http://www.ecocyc.org/**) and WIT (what is there: **http://wit.mcs.anl.gov/WIT2/**). In general, databases designed to contain knowledge on biochemical networks will have a vast amount of different kinds of information about the participants (compounds) of the biochemical reactions. A reaction can mainly be specified in terms of its substrates, products and the enzymes that catalyse it. An enzyme can either be exclusive for a reaction, meaning that they will only catalyse a particular reaction, or specific for a particular type of chemical bond or functional group. In the latter cases, in order to be able to deduce a specific reaction from a general reaction description (referring to compound classes rather than to particular compounds), it is necessary to have a clear classification of chemical compounds. Such a classification is absent, or not explicit, in most pathway databases and, furthermore, most users of the pathway databases tend not to be aware of this problem. There are databases where classification of compounds is incomplete and cannot be used for searching or automatic generation of reactions. The criteria used to classify compounds and pathways also differ from one database to another, and in most cases such criteria are not specified. Figure 1 shows, for example, three different classifications of the Glycolysis pathway in three different databases. During the process of integrating data

Copyright © 2004 John Wiley & Sons, Ltd.

**KEGG**

Metabolism

- Carbohydrate Metabolism

  - Glycolysis /Gluconeogenesis

**EcoCyc**

Energy Metabolism

- Glycolysis

**WIT**

Intermediate Metabolism and Bioenergetics

- Carbohydrate Metabolism

  - Monosaccharides Metabolism

    - Glucose

      - Glucose Catabolism

        - Embden-Meyerhoff-Parnas Pathway

          - EMP From D-glucose

          - EMP From alpha Glucose

          - EMP From beta Glucose

**Figure 1.** Comparison of the classification of the pathway Glycolysis [Embden–Meyerhoff–Parnas (EMP) pathway] used in KEGG, EcoCyc and WIT (Wittig and De Beuckelaer, 2001)

from the different databases, it is necessary to match this information. This is not always a trivial problem to solve, if there is no clear description of the classification criteria applied. Another problem that can be frequently encountered (Wittig and De Beuckelaer, 2001) is the use of compounds with different chemical properties as the same molecule, or identical molecules represented as different compounds.

The aim of our work is to develop methods and tools to support the detection of duplicates, inconsistencies and errors in data related to chemical compounds, biochemical reactions and biochemical pathways. The tools presented in this paper work on the basis of classifications of chemical compounds to detect the problems listed above. Based on the classification of chemical compounds, it is possible to classify biochemical reactions and, in turn, biochemical pathways.

In the available pathway databases reactions are represented on different levels of abstraction. Reaction participants can be referred to as specific compounds (specific molecules) or as general compound classes, which can be subdivided into more specific molecules or molecule classes. We will refer to this last class as general compounds. For example, there are reactions using specific compounds such as *ATP* (*adenosine triphosphate*) as reaction participants, and other reactions defined using more general compounds such as *NTP* (*nucleoside triphosphate*), including all subtypes of *nucleosides* (*ATP, GTP, CTP, UTP*, etc.). In most reactions there is a mixture of specific and general compounds, e.g. $pyruvate + NTP \rightarrow phosphoenolpyruvate + NDP$. *Pyruvate* and *phosphoenolpyruvate* represent specific compounds, whereas *NTP* and *NDP* are general compounds. Since *adenosine* can be classified as a *nucleoside*, and according to that *ATP* is classified as *NTP*, this reaction can be also represented using this specific subtype of *NTP*: $pyruvate + ATP \rightarrow phosphoenolpyruvate + ADP$. Using information about the classification of compounds, it is possible to generate all possible reactions (with specific compounds) that are represented by a general reaction. However, in some cases not all possible subcompounds of a compound class can be participants in a reaction. So, although the reaction is described as involving a participant of a certain type, it is really a subset of the compounds of this type that can act as participants. In this case, it

Copyright © 2004 John Wiley & Sons, Ltd. 

is necessary to look at the chemical structure of the compounds participating. This goes beyond the objective of the tools presented in this paper.

In situations where enzymatic reactions are defined using general compounds, there are also cases where we cannot deduce the specific compounds very easily. For example, under the Enzymatic Classification No. EC 2.7.1.69, the International Union of Biochemistry and Molecular Biology (IUBMB) defined the associated enzymatic reaction as a reaction converting one *sugar* to a *sugar phosphate* catalysed by a *phosphotransferase*. Only in the comments of the enzyme entry is a specification found: '*aldohexoses*, and their *glycosides* and *alditols* are phosphorylated on O-6, *fructose* and *sorbose* on O-1; *glycerone* and *disaccharides* are also substrates' (IUBMB, 1992). Since the compound *sugar* is very general and would include all *carbohydrates*, the comments contain a more detailed definition of *enzyme* and *reaction*. A clear definition of compound properties, relations between different levels of abstractions for compounds and descriptions of reactions enables the possibility of defining more complex queries to metabolic pathway databases, taking the compound classifications into account.

### Methods and results

The software tools were developed in Java, using jdbc to communicate to the local System for the Analysis of Biochemical data (SABIO) database at EML Research gGmbH (Rojas *et al.*, 2002), from which we can extract information about the compounds and their classes. The first tool described here classifies chemical compounds based on their simplified molecular input line entry specification (SMILES) strings. SMILES is a simple, yet comprehensive, chemical nomenclature also used as a data exchange format (Weininger, 1988; Weininger *et al.*, 1989: **http://www.daylight.com/dayhtml/smiles/**). The second tool visualizes the classification results or compound classifications already introduced in the database. Both tools have been integrated into the BioBrowser, a system for the analysis and representation of biochemical pathways and their associated data, developed at the EML and soon to be released (**http://projects.villa-bosch.de/sdbv/projects**). The classification tool can be used independently from a database, whereas the visualization tool needs binary relations between compounds stored in a database to display the classification. Both tools are not directly connected to each other.

### Classification tool

The classification tool is based on methods developed in the CDK (Chemistry Development Kit, Version cdk-20 030 412; Steinbeck *et al.*, 2003). SMILES strings are transferred into a graph structure, which corresponds to the structural formula of the molecule. Subgraph search algorithms are applied to the graph structure instead of searching the string. Whereas a single molecule can be depicted in several ways as a SMILES string, the conversion into a graph results in a unique representation for each molecule. Using SMILES strings, functional groups or specific properties of molecules can be identified. Based on their functional group definitions, compounds can be grouped into different compound classes. Each compound class is defined by specific functional groups or chemical properties such as the number of atoms or functional groups, information about saturation, etc. There are molecules which can be represented in different isomeric forms. An example is the keto–enol tautomerism, where a compound can be available in both keto and enol forms, but a SMILES string can only represent one tautomeric form. Therefore, the definition of functional groups depends on the information given by the SMILES string.

Additionally, for the definition of compound classes, we used information about the nomenclature and the classification of chemical compounds recommended by the International Union of Pure and Applied Chemistry (IUPAC website). These definitions are visible for the user and are highlighted in the SMILES string and within the structural formula. The structural formula, the totals formula and the molecular weight are generated automatically, based on information given by the SMILES string. Figure 2 shows an example of a list of possible classifications of the compound *glucose*. The assignment of chemical compounds to different compound classes in the database is not done automatically or unchecked. Very complex compounds contain lots of functional groups and can be grouped in many different classes. Therefore, a

Copyright © 2004 John Wiley & Sons, Ltd.

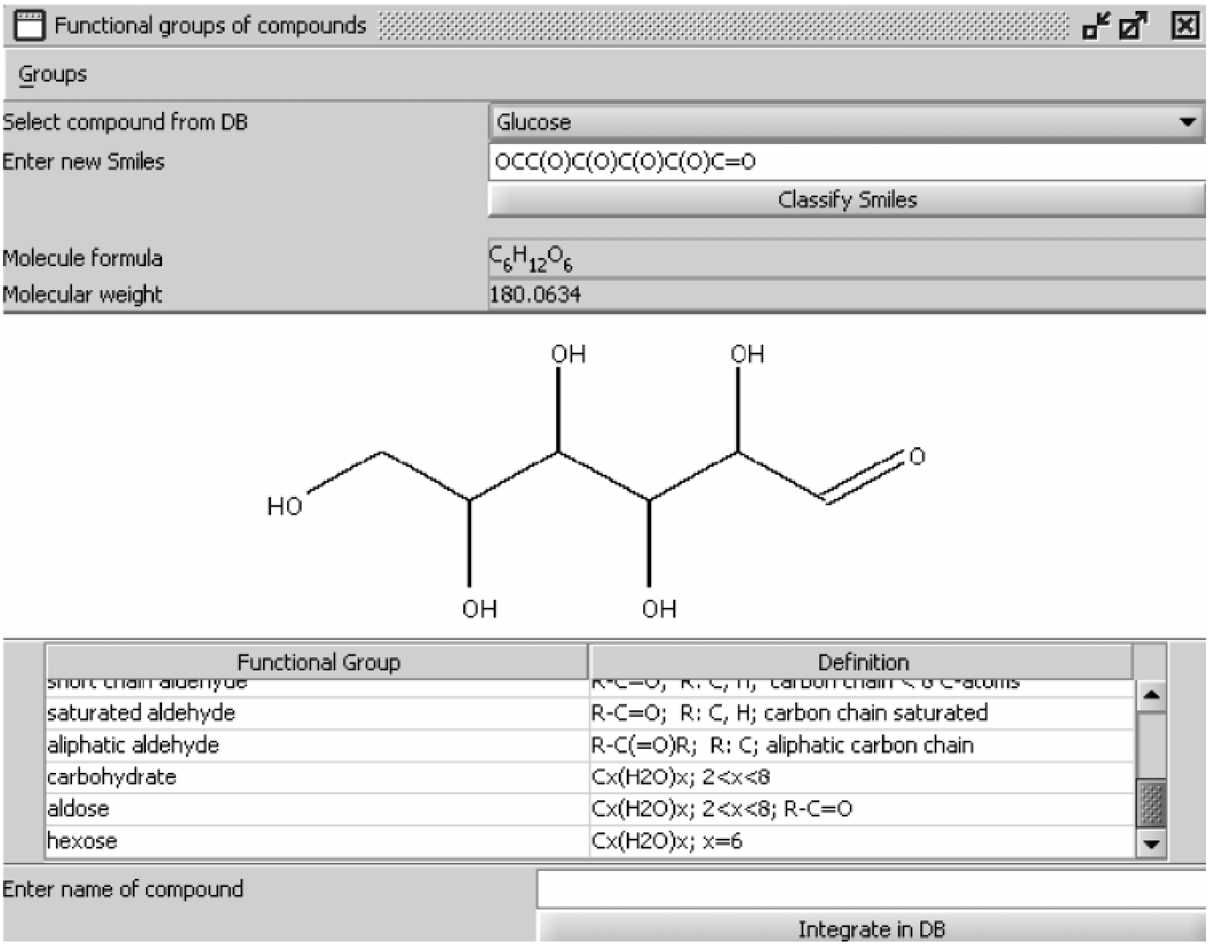

**Figure 2.** Screenshot of the SMILES string-based classification tool using the chemical compound *glucose*

domain expert working as database curator has to decide whether or not all, or only some, of the suggested classifications are useful for biological questions. The resulting classifications of the compound will be incorporated into the database as relationships between the compound and the compound classes. Compound classes are also hierarchically organized, so it is possible to view a compound at different levels of abstraction.

### Visualization tool

The tool for the visualization of classified compounds uses the binary relations between different compound classes or between compound classes and specific molecule entries stored in a database. These binary relations are based on either the classifications of compounds made by the classification tool, or on information extracted from other sources. A compound can be classified in several manners and in turn a compound class can have many compounds or compound subclasses. Of course, no circuits are allowed in the classification (no class can be a subclass or a superclass of itself). The tool presents the tree-like structure of the classifications, showing up to three levels up and/or down in the hierarchy. It is possible to navigate through the tree and to get detailed information about the compounds and their participation in reactions by selecting a compound class or specific compound. Figure 3 shows an example for the graphical representation of the compound g*lucose*, where different classification

Copyright © 2004 John Wiley & Sons, Ltd.

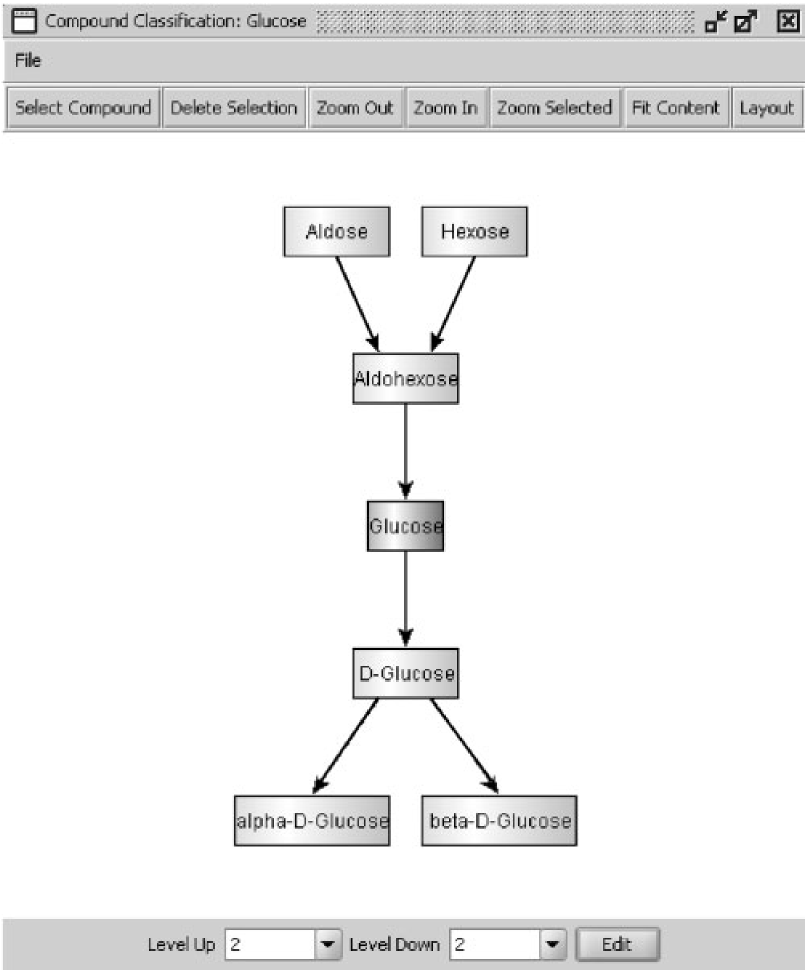

**Figure 3.** Screenshot of the visualization tool using the classification of the chemical compound *glucose* stored in the local pathway database criteria can be observed. *Glucose* is a subtype of the compound class called *aldohexose*, which in turn can be classified as *aldose* and *hexose. Aldoses* are *carbohydrates* containing an *aldehyde* group as the main functional group compared to *ketoses* containing an *oxo* group. On the other hand, g*lucose* is also a *hexose* if the number of C-atoms is used as the classification criterion.

### Relation between reactions and the classification of compounds

The classification results confirmed by the database curators are integrated into our biochemical pathway database and can be used to query the database. Consider the example regarding *NTP*, introduced above. Using the BioBrowser we can execute the following query: 'Find all reactions using *pyruvate* and *NTP* as substrates'. The query result would not only show the reaction $pyruvate + NTP \rightarrow phosphoenolpyruvate + NDP$ but also all reactions, including the compounds which are classified as *NTP*s:

$$pyruvate + ATP \longrightarrow phosphoenolpyruvate + ADP$$

$$pyruvate + GTP \longrightarrow phosphoenolpyruvate + GDP$$

Copyright © 2004 John Wiley & Sons, Ltd. 

$$\textit{pyruvate} + \textit{CTP} \longrightarrow \textit{phosphoenolpyruvate} + \textit{CDP} \text{ etc.}$$

On the basis of the compound classification, biochemical reactions and pathways can be classified into different categories. For example, some reactions of the pathway Glycolysis are using *monosaccharides* as participants, so that this pathway can be classified as *carbohydrate metabolism*. In contrast, selecting the *ATP*-producing reactions of this pathway results in an assignment to *energy metabolism*.

### Discussion

In this paper we have presented two tools, one for the classification of chemical compounds and another for the display of compound classification hierarchies. The classification system offers an automatic and extended categorization of chemical compounds. The classification is performed using different criteria, based on different functional or chemical properties of the compounds. This classification groups compounds participating in reactions into specific or general compounds. The grouping and classification of compounds can be used to classify, group or refine reactions and in consequence the pathways in which they participate. In this way querying for reactions based on a general compound description will return all possible reactions satisfying the classification of the compound and not only the general one. The tool for the visualization of compound classifications uses information extracted from the SABIO database, containing information about biochemical reactions. The tools developed can support the curators of biochemical pathway databases in the processes of data integration and database population. A classification of compounds integrated into a pathway database allows the definition of more complex database queries than those that are possible in most available metabolic pathway databases. For example, based on the recommendations of the IUBMB, many enzymatic reactions are defined as general reactions (in terms of general compounds or compound classes); using the compound classification information, it is possible to get a list of specific compounds being potential participants in these reactions.

The introduction of the tools presented here also helps to improve the reliability of the database, given that it is possible to check for errors and inconsistencies within or between different databases. The classification tool can be used for classifying compounds which have to be included in the database for the first time, for checking existing entries in the database, and to detect errors such as multiple entries for the same compound.

The classification tool will soon be available as a web application independent from a database. For each chemical compound represented as a SMILES string, the tool can be used to suggest possible classifications of the compound. Some currently available pathway databases (e.g. WIT and EcoCyc) offer compound information including SMILES for a list of compounds. These data could be used for a classification and the classification results could be integrated into a local database, dependent on the database schema.

Both tools are independent from each other but once the results of the compound classification have been stored in the database, it is possible to use the visualization tool to view the resulting classification.

### Acknowledgements

This project was supported by the Klaus Tschira Foundation (KTF). The authors would also like to thank Andreas Eberhart for his contribution in the integration of the tools to the BioBrowser system.

### References

IUBMB, (International Union of Biochemistry and Molecular Biology). 1992. *Enzyme Nomenclature: Recommendations of the Nomenclature Committee of the IUBMB*. Academic Press: New York.

IUPAC website: **http://www.chem.qmul.ac.uk/iupac/**

Rojas I, Bernardi L, Ratsch E, Kania R, Wittig U, Saric J. 2002. A database system for the analysis of biochemical pathways. *In Silico Biol* **2**(2): 75–86.

Steinbeck C, Han Y, Kuhn S, *et al.* 2003. The Chemistry Development Kit (CDK): an open-source Java library for chemo- and bioinformatics. *J Chem Inf Comput Sci* **43**(2): 493–500.

Weininger D. 1988. SMILES, a chemical language and information system. 1. Introduction to methodology and encoding rules. *J Chem Inf Comput Sci* **28**: 31–36.

Copyright © 2004 John Wiley & Sons, Ltd. 

Weininger D, Weininger A, Weininger JL. 1989. SMILES. 2. Algorithm for generation of unique SMILES otation. *J Chem Inf Comput Sci* **29**: 97–101.

Wittig U, De Beuckelaer A. 2001. Analysis and comparison of metabolic pathway databases. *Brief Bioinform* **2**(2): 126–142.

Copyright © 2004 John Wiley & Sons, Ltd. 
